# Control of CD56 expression and tumor cell cytotoxicity in human Vγ2Vδ2 T cells

**DOI:** 10.1186/1471-2172-10-50

**Published:** 2009-09-21

**Authors:** Elizabeth M Urban, Haishan Li, Cheryl Armstrong, Chiara Focaccetti, Cristiana Cairo, C David Pauza

**Affiliations:** 1 Institute of Human Virology, University of Maryland School of Medicine, Baltimore, MD, 21201, USA; 2 Graduate Program in Microbiology and Immunology, University of Maryland, Baltimore, MD; 3 University of Rome Tor Vergata, Rome, Italy

## Abstract

**Background:**

In lymphocyte subsets, expression of CD56 (neural cell adhesion molecule-1) correlates with cytotoxic effector activity. For cells bearing the Vγ2Vδ2 T cell receptor, isoprenoid pyrophosphate stimulation leads to uniform activation and proliferation, but only a fraction of cells express CD56 and display potent cytotoxic activity against tumor cells. Our goal was to show whether CD56 expression was regulated stochastically, similar to conventional activation antigens, or whether CD56 defined a lineage of cells committed to the cytotoxic phenotype.

**Results:**

Tracking individual cell clones defined by their Vγ2 chain CDR3 region sequences, we found that CD56 was expressed on precursor cytotoxic T cells already present in the population irrespective of their capacity to proliferate after antigen stimulation. Public T cell receptor sequences found in the CD56+ subset from one individual might appear in the CD56- subset of another donor. The commitment of individual clones to CD56+ or CD56- lineages was stable for each donor over a 1 year interval.

**Conclusion:**

The ability to express CD56 was not predicted by TCR sequence or by the strength of signal received by the TCR. For γδ T cells, cytotoxic effector function is acquired when cytotoxic precursors within the population are stimulated to proliferate and express CD56. Expression of CD56 defines a committed lineage to the cytotoxic phenotype.

## Background

Potent effector cells among CD8+ αβ T cells, NK cells, NKT cells and γδ T cells each have specific mechanisms for identifying malignant or infected targets and sparing normal cells. Common to all is the expression of CD56 (neural cell adhesion molecule 1; NCAM-1) on the effector cell subset [1-5]. For example, CD56+ CD8+ T cells with high expression of perforin and Granzyme B are potent cytotoxic effectors that possess an oligoclonal Vβ chain repertoire [2]. NK cells lack T cell receptors (TCR) and rely instead on complex families of NKR and KIR molecules to identify target cells [6,7]. The NKT cells express an invariant Vα24 chain that is responsible for their CD-1 restricted responses to Galactosyl ceramide [8,9].

We are trying to understand tumor cell recognition by human Vγ2Vδ2 T cells (also called Vγ9Vδ2 in an alternate nomenclature). This population is dominated by cells expressing the Vγ2-Jγ1.2 rearrangement which endows the MHC-unrestricted response to isoprenoid pyrophosphates (phosphoantigens) or synthetic aminobisphosphonates that cause accumulation of phosphoantigens, and specific human tumor cell lines [10-13]. Despite complexity of the Vγ2 chain CDR3 region [14-16], individual cell clones respond similarly to phosphoantigens or tumor cells [13] and it remains unclear how activated Vγ2Vδ2 cells discriminate normal from malignant cells and what controls cytotoxicity.

Recently, we [17] reported that CD56 was expressed on a potently cytotoxic subset of human Vγ2Vδ2 T cells, similar to what was reported for CD8 T cells [2]. However, strong proliferation responses to phosphoantigen occurred in both CD56+ and CD56- populations. This highly selected oligoclonal Vγ2Vδ2 + population responds uniformly to phosphoantigen, yet only ~50% of expanded cells express CD56 and lyse tumor cell targets. We imagined three alternative models to explain these observations: 1) CD56 expression is regulated stochastically perhaps by cytokine signals; 2) Vγ2Vδ2 T cell receptors vary in strength or specificity of antigen binding and these properties are related to control of CD56 expression; 3) CD56 is expressed only on CTL precursors that represent distinct subsets or lineages.

Our initial approach for defining the CD56+ subpopulation used Vγ2 sequences as markers for individual T cell clones. Vγ2Vδ2 T cell lines were generated from PBMC by phosphoantigen treatment and separated into CD56+ and CD56- fractions. A sample of Vγ2 chains from each fraction was cloned and sequenced. If the same Vγ2 sequences are detected in both fractions, expression of CD56 must be regulated stochastically, in accordance with model 1 (above). If the Vγ2 chain repertoire is distinct between the CD56+ and CD56- fractions, we need to test whether a previously missed antigen specificity within the Vγ2Vδ2 repertoire dictates CD56 expression and tumor cell cytolysis. For testing the latter, we can exploit the observation that human Vγ2 repertoire has a high proportion of public clonotypes with complete CDR3 region identity among unrelated individuals [13]. By comparing phenotypes for cells expressing public clonotypes, in terms of whether they express CD56 similarly among unrelated individuals, we learn whether TCR specificity dictates CD56 expression.

Our results argue that individual Vγ2Vδ2 T cell clones are already committed to either the CD56+ or CD56- subset. Commitment is not due to special properties of the TCR but seems, within the limits of our analysis, to be a property distinct to each clone. The circulating Vγ2Vδ2 T cell pool is the result of chronic, positive selection [18] and individual clones seem to be long-lived--at least 7 years in adults [13]. Thus, the mechanisms controlling CD56 expression can have long-term impact on the capacity for tumor cell cytotoxicity.

## Methods

### PBMC Isolation, *In vitro *IPP stimulation and expansion of Vγ2Vδ2 T cells

Whole blood was obtained with written consent from healthy human volunteers and approved by the Institutional Review Board at the University of Maryland School of Medicine. Donors studied here are representative of the specimen collection in our laboratory. Total lymphocytes were separated from heparinized peripheral blood by density gradient centrifugation (Ficoll-Paque; Amherson Biosciences, Piscataway, NJ). Isolated PBMC were then cultured at 1 × 10^6 ^cells/ml in RPMI-1640 media supplemented with 10% fetal bovine serum (FBS), 100 U/ml penicillin and 100 U/ml streptomycin (GIBCO, Grand Island, NY) and 100 U/ml recombinant human interleukin-2 (rIL-2) (Tecin, Biological Resources Branch, NIH, Bethesda, MD). To stimulate PBMC, 15 μM of isopentenyl pyrophosphate (IPP) (Sigma, St. Louis MO) were added. Cultures were incubated for 14 days at 37°C with 5% CO_2 _and media with IL-2 was replenished every 3-4 days. Cell proliferation was confirmed by flow cytometry using monoclonal antibodies specific for Vγ2 (FITC), CD56 (PE), CD3 (APC), and isotype-matched, IgG controls. At least 1 × 10^4 ^lymphocytes were analyzed per sample on a FACSCalibur flow cytometer (BD Biosciences, San Diego, CA). Flow cytometry data were analyzed using FlowJo software.

### Cell Separation and DNA Synthesis

Fresh PBMC and cell lines obtained after IPP stimulation were separated into CD56+ and CD56- populations using magnetic bead techniques. Specifically, 1 × 10^7 ^to 2 × 10^7 ^cells were treated with anti-CD56 conjugated to PE (Beckman Coulter, Inc) at 15 μl/10^7 ^cells, followed by magnetic-bead labeled anti-PE (Miltenyi Biotec., Auburn, CA) at 30 μl/10^7 ^cells. Labeled cells were passed through a magnetic column and fractions were collected. This procedure was repeated to achieve highest possible purity. Total RNA was extracted from at least 1 × 10^6 ^CD56+ or CD56- cells using RNeasy Mini Kits (Qiagen, Valencia, CA) according to the manufacturer's instructions. One μg of total RNA was converted to cDNA using a reverse transcription kit (Promega, Madison, WI). Each reaction was incubated at 42°C for 120 min and then diluted with RNAse/DNase free H_2_O to a total volume of 100 μl. Five μl of cDNA served as the template for subsequent PCR reaction to amplify the Vγ2 T cell chain using 2.5 μM Vγ2 forward primer (5'-atcaacgctggcagtcc-3') and 2.5 μM Cγ1 reverse primer (5'gttgctcttcttttcttgcc-3') under the following cycling conditions: 5 min denaturation at 94°C, 30 cycles of 1 min at 94°C, 1 min at 60°C, and 1 min at 72°C, followed by a final elongation for 10 min at 72°C AmpliTaq Gold (Applied Biosystems, Inc., Branchburg, New Jersey).

### Cloning and Sequencing

PCR products were separated on 1% agarose gels and purified using a QIAquick gel extraction kit (Qiagen) according to the manufacturer's instructions. Purified products were denatured for 1 min at 94°C, incubated for 30 min at 72°C with 2 mM MgCl2, 0.2 mM dATPs, 2.5 U AmpliTaq Gold, then ligated into the pCR2.1 cloning vector. The ligated vector was transfected into TOP10F' competent E. coli cells (TA Cloning Kit, Invitrogen, Carlsbad, CA) and bacterial colonies were grown overnight on agar plates containing 50 μg/ml ampicillin, 500 μM isopropylthiogalactoside (IPTG) and 80 μg/ml 5-bromo-4-chloro-3-indolyl-B-D-galactopyranoside (X-Gal). White colonies, containing the recombinant plasmid, were cultured overnight in LB supplemented with 50 μg/ml ampicillin, then plasmid DNA was purified using Real 96 Miniprep kits (Qiagen). Sequencing reactions used the Big Dye v3.1 fluorescent sequencing kit (Applied Biosystems, Foster City, CA) with the M13 forward and reverse oligonucleotide primers. Sequences were analyzed on an ABI3700 sequencer and aligned using Sequencher and MacVector software. In total, approximately 5,000 Vγ2 sequences were annotated, aligned and compared during this study.

### Daudi Cultures

The B cell lymphoma cell line, Daudi, was obtained from ATCC (CCL-213). Cells were cultured at 37°C with 5% CO_2 _at a concentration between 3.5 × 10^5 ^to 1 × 10^6 ^cells/ml. Cultures were kept in RPMI-1640 media supplemented with 10% FBS, PCR extension temp to 72°, 2 mM L-glutamate, 4.5 g/L glucose, 1.5 g/L NaHCO_3_, 10 mM HEPES, and 1 mM sodium pyruvate (GIBCO). Cells were cultured for no more than 20 passages.

### γδ T Cell Cloning

γδ T cells clones were obtained following a previously published procedure [15], with some modifications. Specifically, PBMC were isolated from whole blood as described above. Using the MACS system and the Vγ2 monoclonal antibody conjugated to PE, we isolated the Vγ2 + fraction of cells. The cells were incubated in media overnight at 37°C with 5% CO_2_. In a 96-well V-bottom microtiter plate (Corning, NY), 1 cell/well of the Vγ2+ population was obtained by limiting dilution and combined with 1 × 10^5^/well irradiated (5000 rad) autologous PBMC and 1 × 10^4^/well irradiated (5000 rad) Daudi in a total volume of 100 μl. 15 μM IPP and 100 U/ml IL-2 was also added to each well. The plates were incubated for 21 days at 37°C with 5% CO_2_. Wells that showed signs of cell proliferation were analyzed by flow cytometry for expression of Vγ2, Vδ2, CD56, NKG2D, and NKG2A (BD Biosciences). Clonality of the population was determined by sequencing of the Vγ2 CDR3 region.

### Flow Cytometric Cytotoxicity Assay

Due to the low cell numbers generated by cloning, and the fragility of the cloned γδ T cells when re-expanded, cytotoxicity of the individual γδ T cell clones was measured using a flow cytometry based assay. In this assay, 1 × 10^3 ^Daudi target cells were labeled with CFSE (Cell Trace™, Invitrogen) and then combined in a 96-well microtiter plate (Corning) with Vγ2 cell effectors at effector to target ratios varying from 10:1 to 0.1:1. Reactions to control for spontaneous lysis included only the CFSE-labeled targets and medium. Plates were incubated at 37°C with 5% CO_2 _for 4 hours. After the incubation, 10 ul of 18% paraformaldehyde in phosphate-buffered saline were added to inactive effector cells, then we added 2.5 × 10^3 ^Daudi cells that had been labeled with PKH26 (Sigma Corp., St. Louis, Missouri). Cells were then analyzed by flow cytometry, measuring the proportions of green (CFSE) and red (PKH26) labeled Daudi cells. A decrease in the proportion of green:red indicated cytotoxicity of the added Vγ2+ cells. Specific lysis was determined by comparing the ratio of PKH26-labeled reference cells with CFSE-labeled target cells in the following formula: (CFSE:PKH26 control - CFSE target:PKH26 reference) × 100.

## Results

### Asymmetric CD56 expression in Vγ2Vδ2 T cells

**Figure 1:**
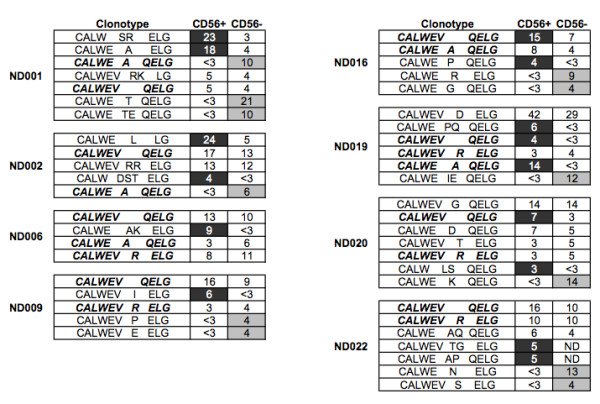
**Asymmetric CD56 Expression in Vγ2Vδ2 T cells**. IPP-stimulated cell lines were generated from 8 normal human donors and fractionated by magnetic bead separation into CD56+ and CD56- subsets. Vγ2 chains from each fraction were sequenced and translated. The frequency of each chain, identified by their unique CDR3 regions, with the fraction was calculated. Clonotypes found at a frequency below 3% were below the levels of detection for this assay, since below that cutoff we cannot reproducibly detect them. Clonotypes were considered committed to either the CD56+ or CD56- populations if the distribution was greater than 2:1. Black shading identifies clonotypes committed to CD56+ population, while those committed to the CD56- population are shaded in gray.

The Vγ2Vδ2+ cell lines obtained after phosphoantigen stimulation of PBMC contain CD56+ and CD56- subsets. These subsets were separated by magnetic bead sorting and the TCR repertoire was characterized by cloning and sequencing a sample of the Vγ2 chains in each fraction. The DNA sequences were aligned and translated into amino acid sequences representing the individual clonotypes found for each donor. The most abundant clonotypes from each donor and whether they appear in CD56+ or CD56- fractions are shown (Figure 1).

As expected, we found both private (unique to one donor) and public (found in more than one donor) clonotypes among these Vγ2 chains. Public clonotypes match at all positions including the CDR3 region encoded partly by non-templated nucleotides. Public Vγ2 clonotypes found in these data sets include WEV---QELG, WEV---R---ELG and WE---A---QELG. We refer to WEV---QELG as the "canonical" clonotype because it is mostly encoded by germline sequences without deletion of V or J segments and without nucleotide additions. Among 8 donors, public Vγ2 clonotypes appeared 18 times. In 12 of 18 examples, the clonotypes appeared in both CD56+ and CD56- fractions with < 2-fold difference in frequency between the 2 fractions. In the remaining 6 of 18 (33%) appearances, the public clonotype was more abundant in either CD56+ or CD56- fractions--a condition referred to as asymmetry. Among these 8 donors, we found 28 highly abundant private Vγ2 clonotypes. Of the private clonotypes, 21 (75%) were distributed asymmetrically (>2-fold difference).

These data suggested that public clonotypes were associated randomly with CD56+ or CD56- fractions or distributed symmetrically, but private clonotypes were distributed asymmetrically into the two subsets. However, only a single sample from each donor was analyzed, so we could not determine if the association with CD56 expression was stable. To address this issue, we needed to concentrate on a smaller number of donors to complete more extensive studies including replicate sampling. We selected donors ND001, ND006 and ND019 as representatives of the donor pool.

### Stably expressed Vγ2 chain sequences can be used to compare CD56+ and CD56- subsets

We compared Vγ2 clonotype (the amino acid sequence for the Vγ2 chain CDR3 region) frequencies among three healthy donors in samples collected approximately 1 year apart using 200-300 Vγ2 chain cDNA clones from each specimen. The Vγ2 chain cDNA from PBMC or IPP-stimulated cell lines were cloned into plasmids and sequenced individually. The frequencies for individual Vγ2 clonotypes were calculated as a proportion of the total number of sequences obtained from each cell sample. Only clonotypes found at a frequency of 3% or greater were studied; below that level, frequency values are less reproducible due to sampling errors. Clonotypes detected in IPP-expanded lines from both 2007 and 2008 time points were also deemed stable. Frequencies for each stable clonotype from three donors ND001, ND006, and ND019 are shown (Table 1). In each case, the proliferation response to IPP was similar at both time points for each clonotype.

**Table 1:** Stable Clonotypes.

**Clonotypes**	**Ex Vivo**		**IPP Expand**	
**DONOR: ND001**	**2007**	**2008**	**2007**	**2008**

CALW SR ELG	52	34	13	21

CALWE A ELG	14	28	11	14

CALWE RK QELG	<3	<3	6	3

CALWEV RK LG	3	6	5	9

CALWEV QELG	4	<3	5	4

CALWE T QELG	<3	<3	11	21

CALWE TE QELG	<3	<3	6	10

*N*	*165*	*158*	*151*	*239*

				

**DONOR: ND006**				

CALWEV QELG	11	13	11	13

CALWE AK ELG	<3	9	5	10

CALWE A QELG	11	6	5	12

CALWEV R ELG	<3	4	6	4

*N*	*156*	*206*	*186*	*287*

				

**DONOR: ND019**				

CALWEV D ELG	28	36	36	23

CALWE PQ QELG	10	5	4	7

CALWEV QELG	7	9	4	7

CALWEV R ELG	6	3	3	8

CALWE A QELG	8	8	9	5

CALWE IE QELG	<3	<3	6	5

*N*	*163*	*256*	*166*	*246*

Vγ2 chain clonotypes (CDR3 region) are shown for donors ND001, ND006 and ND019. For each clonotype, we show the proportion of all Vγ2 chains represented by that clonotype for specimens collected in 2007 or 2008. Clonotype abundance for ex vivo and post-expansion samples are shown. The *N *values represent the total number of Vγ2 chain DNA sequences obtained for each specimen.

### CD56 positive and negative fractions from individual donors contain distinct Vγ2 clonotypes

We next measured the frequencies for individual clonotypes in CD56+ and CD56- subsets. PBMC collected 1 year apart and the resulting IPP-expanded cell lines were fractionated into CD56+ and CD56- populations using magnetic bead cell separation. The Vγ2 chain mRNAs were amplified by PCR, cloned in plasmid vectors and sequenced to determine the frequency of individual Vγ2 clonotypes within the population.

**Figure 2:**
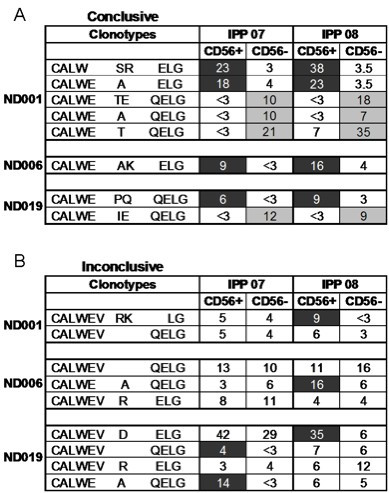
**Conclusive and Inconclusive Clonotypes**. *Ex vivo *PBMC and IPP-expanded cell lines were fractionated by magnetic bead cell separation into CD56+ and CD56- populations. RNA was extracted and converted to DNA. Vγ2 chains were sequenced and the frequency of clonotypes within each population was calculated. Limit of detection of this assay was >3%. Clonotypes were considered committed to either the CD56+ or CD56- populations if the distribution was greater than 2:1 (Conclusive); the remaining clones were considered Inconclusive as there was no clear bias in their distribution. Black shading identifies clonotypes committed to CD56+ population, while those committed to the CD56- population are shaded in gray.

The most common Vγ2 clonotypes from donors ND001, ND006, and ND019 are shown (Figure 2A). If a particular clonotype was more abundant in one fraction by a difference greater than 2:1, it was assigned as CD56+ or CD56- as appropriate. Clonotype distributions varied among donors. In ND001, 5 out of 7 (71.4%) stable clonotypes expanded preferentially into either CD56+ or CD56- fractions. In ND006 and ND019, 1 of 4 (25%) and 2 of 6 (33.3%) stable clonotypes showed this asymmetry. The patterns for clonotype distribution were consistent over the 1-year sampling period. For example, the ND001 clonotype CALW---SR---ELG was 8 times more frequent in the 2007 CD56+ fraction and was 10 times more frequent in 2008. CALWE---TE---ELG was in the CD56- population at similar frequencies for both time points.

**Figure 3:**
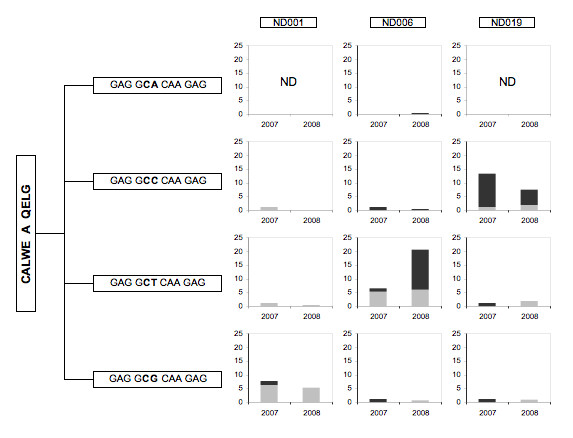
**Multiple Vγ2 nucleotypes comprise individual public clonotypes**. Public clonotypes are usually encoded by more than one circulating nucleotype. The amino acid sequences appear in the vertical box at the left. The possible nucleotypes that encode this clonotype are shown in the horizontal boxes as nucleotide sequences; the hypervariable or N nucleotides are shown in bold type. The histograms show the abundance of each nucleotype (as a proportion of all sequences in the CD56+ or CD56- samples) among IPP-expanded cell lines generated from PBMC collected in 2007 or 2008. The bar height represents the total nucleotype abundance; the dark shaded portion indicates the fraction of sequences in the CD56+ subset and the light shaded region shows the fraction of nucleotypes in the CD56- subset. The label "ND" indicates that nucleotype was "not detected" in the 2007 or 2008 cell lines.

**Figure 4:**
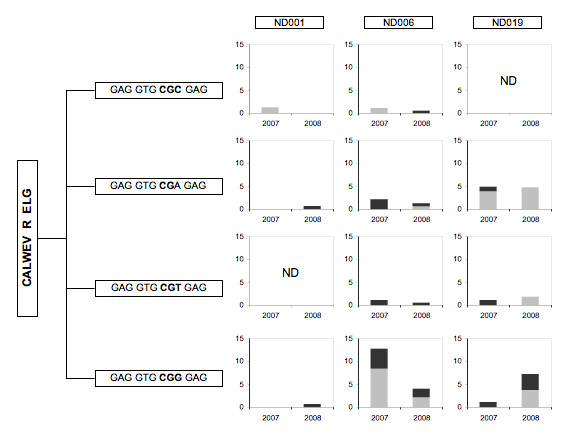
**Multiple Vγ2 nucleotypes encode the public clonotype CALWEV---R---ELG**. Nucleotypes encoding the public clonotype CALWEV---R---ELG are listed. Histograms show the abundance of each nucleotype in two distinct samples with dark shading indicating the proportion of CD56+ sequences and the light shading showing sequences in the CD56- fraction (as described for Figure 3). ND indicates a particular nucleotype was not found for those specimens.

**Figure 5:**
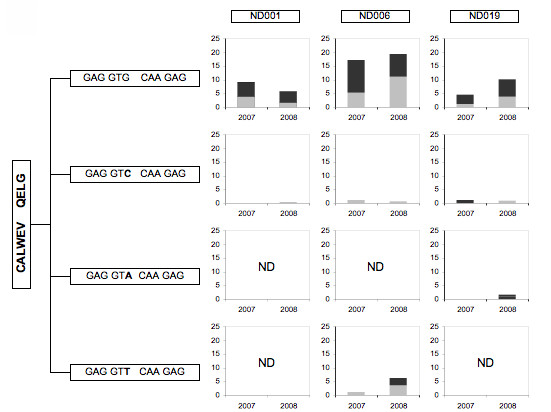
**Multiple nucleotypes encoding the canonical Vγ2 chain CALWEV---QELG**. The clonotype CALWEV---QELG is designated as the canonical sequence because it can be expressed with N-region additions. However, this public clonotype is also encoded by multiple nucleotypes as shown here. Histograms show the proportion of each nucleotype in the total sequence set (bar height) for two distinct samples. Dark shading indicates the proportion of sequences in CD56+ cells while light shading refers to the proportion of sequences found in CD56- cells. ND indicates that a particular nucleotype was not found for those samples.

We next looked at clonotypes where there was no clear bias toward CD56+ or CD56- fractions (Figure 2B), the so-called "inconclusive" clonotypes. Many of these are public clonotypes that is, they are present in more than one donor. In our experience, public clonotypes are usually encoded by more than one nucleotide sequence (nucleotypes). We examined Vγ2 nucleotypes for each public clonotype and calculated their respective frequencies (Figures 3, 4 &5). Nucleotype analysis explained the behavior for some of the inconclusive clonotypes. For example, the ND019 clonotype CALWE---A---QELG was skewed to the CD56+ population in a 2007 IPP-expanded specimen, but was less biased in a 2008 sample (Figure 2B). Singling out its most abundant nucleotype, GCC---CAA, we see it was skewed to the CD56+ population in both samples at frequencies of 12% to < 3% and 6% to < 3%, respectively (Figure 3). Clearly, this nucleotype was committed to the CD56+ phenotype even if the overall clonotype appeared unbiased. Other nucleotypes (GCT---CAA and GCG---CAA) behaved differently in the 2007 and 2008 specimens causing them to appear inconclusive i.e., observable in both CD56+ and CD56- fractions. The ND019 clonotype CALWEV---R---ELG was found in both CD56+ and CD56- populations. However, we identified a major nucleotype, GTG---CGA that was more common in the CD56- population (Figure 4). Again the distinct behavior of individual nucleotypes confused the clonotype analysis. We cannot always analyze the distribution of every nucleotype within a public clonotype because some are infrequent, but this level of analysis supported the view that CD56 expression is not a random marker and most of the individual nucleotypes each have distinct expression of CD56.

Nucleotype analyses explained most but not all of the inconclusive clonotypes. The public canonical sequence, CALWEV---QELG, is the Vγ2-Jγ1.2 germline sequence without junctional nucleotides. It was not consistently segregated into CD56+ or CD56- fractions for any donor at the clonotype (Figure 2B) or nucleotype (Figure 5) levels. Early γδ cell ontogeny is characterized by a high frequency of canonical recombinations [19]. Therefore, the presence of this Vγ2 chain sequence does not imply clonality because it likely arises again and again throughout life. Other inconclusive clonotypes, such as CALWEV---D---ELG and CALWEV---RK---LG were distributed differently in 2008 compared with the 2007 specimens. These clonotypes are each encoded by a single nucleotype, implying they represent a single clone of cells. Whether clones can change in terms of the capacity to express CD56 or whether new clones arose with the same nucleotype, cannot be discerned from our data. However, most of the clones we observed had the same phenotype in PBMC collected 1 year apart and the capacity for CD56 expression appears to be stable.

### Identical Vγ2 nucleotypes are present among unrelated donors

We observed a number of identical Vγ2 chains among unrelated donors. For example, the nucleotype GTG---CGG---GAG was in all 3 donors (Figure 4). In ND006, it was the most abundant nucleotype encoding the public clonotype CALWEV---R---ELG. In donor ND019, it was equally abundant as the GTG---CGA---GAG nucleotype. Individual nucleotypes encoding the public clonotype CALWE---A---QELG were also found in multiple donors (Figure 3). The canonical nucleotype (GTG---CAA) was abundantly present in all 3 donors, but this was not surprising, given the high frequency of canonical recombinations during ontogeny. Interestingly, the canonical clonotype (WEV---QELG) was not always the product of germline sequences, as non-canonical nucleotypes also encoded this chain (Figure 5).

### Vγ2 chain sequence did not predict CD56 expression

We evaluated in three donors whether public clonotypes appear consistently in either CD56+ or CD56- subsets. The clonotype CALWE---A---QELG is a useful example (Figure 3). In ND001, this clonotype (represented by the dominant nucleotype GCG---CAA) was predominantly in the CD56- population. In ND019 the dominant nucleotype (GCC---CAA) was in the CD56+ population. ND006 is unusual in that the clonotype's dominant nucleotype (GCT---CAA) was in the CD56- population of the 2007 sample and in the CD56+ population of 2008. This suggests that individual clones can switch to a different committed phenotype or a new clone arose with the same CDR3 region sequence. Since we have observed identical nucleotypes in unrelated donors, it is assumed that the same sequences can be generated more than once in a single donor and we expect to find some nucleotypes with inconsistent behavior.

Our data suggest that the ability of Vγ2Vδ2 T cells to express CD56 can be separated from the strength of proliferative responses received through the TCR. After expanding the Vγ2Vδ2 subset by IPP, the ND001 clonotype CALW--SR---ELG increased to 21.3% of the Vγ2 positive population (Table 1). The CALWE---T---QELG expressing cells also increased to a frequency of 21%. However, the expanded CALW---SR---ELG cells expressed CD56, and the CALWE---T---QELG cells remained CD56 negative (Figure 2A). We have observed examples of rapidly proliferating cells in both CD56+ and CD56- subsets and strength of response to phosphoantigen does not appear to influence CD56 expression.

### IL2 alone induces CD56 expression, but the repertoire is distinct from phosphoantigen expanded cell lines

We noted that culture with IL-2 alone did not expand the Vγ2+ cells (Figure 4A) but did induce CD56 expression on Vγ2Vδ2 cells (Figure 4B). IL-21, a cytokine that enhances both proliferation and effector functions of αβ T cells and another member of the common γ chain cytokines, also induced CD56 expression on γδ T cells [20]. This suggests that signaling through the Vγ2Vδ2 TCR is not required for expression of CD56 and encouraged us to understand the clone complexity of IL-2-treated CD56+ cells. We compared the Vγ2 repertoire of CD56-depleted PBMC treated with IL-2 alone to the IPP-expanded cell lines to test this hypothesis.

**Figure 6:**
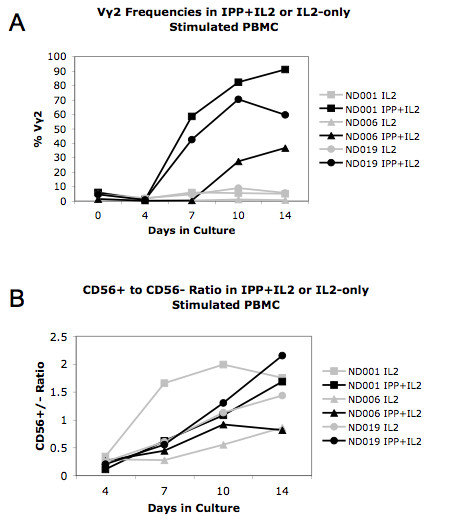
**IL-2 induces expression on Vγ2+ cells without expansion**. *Ex vivo *PBMC were collected from our 3 donors and depleted of CD56+ cells by magnetic cell separation. Samples were cultured for 14 days with IPP+IL-2 or IL-2 alone. Cells were collected from the cultures at regular intervals and analyzed for Vγ2 and CD56 expression by FACS. The percentage of Vγ2+ cells in the cell lines are shown in Figure 6A. The ratio of CD56+ Vγ2+ to CD56- Vγ2- cells was calculated and plotted in Figure 6B. Samples that were treated with IL-2 alone are shown in gray, while black markings indicate IPP-expanded cultures. We also measured changes in Vγ2+ cells during 14 days after IPP+IL-2. For ND001, Vγ2+ cells went from 1.6 × 10^7 ^to 9.1 × 10^7 ^cells (5.7 fold increase), for ND006 the increase was from 1.2 × 10^7 ^to 6.7 × 10^7 ^cells (5.6 fold increase) and for ND019 the increase was from 1.8 × 10^7 ^to 8.4 × 10^7 ^(4.7 fold increase).

We depleted CD56+ cells from PBMC with magnetic bead separation. The remaining CD56- cells were treated with IL-2. The IL-2 alone caused little to no proliferation of the Vγ2+ population. In contrast, IPP + IL-2 stimulation caused significant expansion, with Vγ2+ cells making up 40 to 90% of PBMC by 14 days after stimulation (Figure 6A). Despite the lack of significant expansion, by day 14 the IL-2-only treated Vγ2 cells had increased expression of CD56 (Figure 6B). We next tested whether similar clonotypes were present in CD56+ or CD56- fractions after IL-2 alone compared to IPP + IL-2 stimulation.

Clonotype frequencies were starkly different for the IL-2 treated PBMC compared with cell lines obtained after IPP + IL-2 (Table 2). CALWEV---D---ELG was the dominant clonotype in PBMC from ND019 and IPP-expanded Vγ2 cells with frequencies of 36% and 23% respectively. In the IL-2-only culture, the frequency of this clonotype dropped to 2%. In ND006, a dominant clonotype CALWEV---QELG, fell from 13.4% in PBMC, to < 3% in the IL-2-only culture. For ND006, the total frequency of peripheral Vγ2-Jγ1.2 T cells dropped from 87% to 17.4% following IL-2 treatment (data not shown). For ND001, the dominant PBMC clonotypes, CALW---SR---ELG and CALWE---A---ELG, were still present at high frequencies after IL-2 but other IPP-inducible clonotypes including CALWEV---QELG, CALWE---T---QELG and CALWE---TE---QELG were distinctly low after IL-2 treatment.

**Table 2:** Vγ2 clonotype frequencies in IL-2 treated cultures.

**Clonotypes**	**Ex Vivo**	**IPP**	**IL2**
**DONOR: ND001**	**2008**	**2008**	**2008**

CALW SR ELG	34	21	25

CALWE A ELG	28	14	34

CALWE RK QELG	6	9	<3

CALWEV RK LG	<3	4	<3

CALWEV QELG	<3	3	<3

CALWE T QELG	<3	21	<3

CALWE TE QELG	<3	10	<3

*N*	*158*	*239*	*268*

			

**DONOR: ND006**			

CALWEV QELG	13	13	<3

CALWE AK ELG	9	10	<3

CALWE A QELG	6	12	<3

CALWEV R ELG	4	4	<3

*N*	*206*	*287*	*172*

			

**DONOR: ND019**			

CALWEV D ELG	36	23	<3

CALWE PQ QELG	5	7	<3

CALWEV QELG	8	5	6

CALWEV R ELG	<3	8	9

CALWE A QELG	9	7	18

CALWE IE QELG	<3	5	<3

*N*	*256*	*246*	*242*

Vγ2 chains from donor PBMC, IPP-stimulated cell lines, and IL-2 only treated cells were sequenced. Individual Vγ2 clonotypes frequencies for stable clonotypes were calculated as a proportion of total sequences obtained from each donor sample. Any sequences present at a frequency below 3% are under the limits of detection for this assay. The total numbers of sequences analyzed for each sample are listed.

### CD56+ Vγ2Vδ2 cells clones are cytotoxic for Daudi cells

So far, studies have focused on the Vγ2 repertoire as a marker for clonality. Next, we examined individual biological clones of Vγ2Vδ2 T cells to confirm the link between CD56 expression and cytotoxicity.

**Figure 7:**
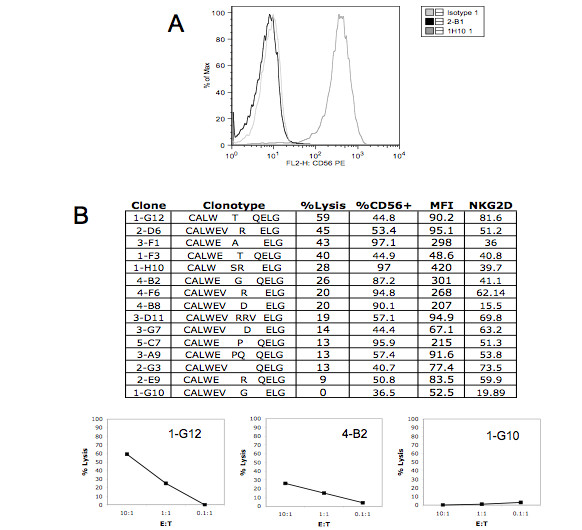
**Vγ2 T cell clones expressing CD56 are cytotoxic against Daudi cells**. γδ T cell clones were generated expansion of a single cell by phosphoantigen in the presence of irradiated feeder cells. Expression of the Vγ2 chain of the TCR, CD56, and NKG2D were determined by flow cytometry. Figure 7A shows CD56 staining of a CD56+ Vγ2+ clone versus a CD56- Vγ2+ clone. Cytotoxicity of each clone was analyzed using a flow cytometry-based assay. Target Daudi cells were labeled with CFSE and cultured with cloned γδ cells at Effector:Target ratios of 10:1, 1:1, and 0.1:1. After 4 hours of incubation, the reactions were stopped with paraformaldehyde and PKH26-labeled Daudi cells were added to each tube. Specific lysis was calculated using the following formula: (CFSE-labeled control ÷ PKH26-labeled control) - (CFSE-labeled target ÷ PKH26-labeled reference) × 100. Assays were performed with triplicate samples and standard deviation was < 15% of the mean. The specific lysis values at E:T of 10:1 are listed for each clone. Cytotoxicity panels for 3 representative clones showing high, medium, and low/no lysis are also shown.

Our initial efforts to obtain clones used PHA stimulation followed by limiting dilution on feeder cells [15]. We acquired a small number of clones with this method--9/27 being CD56+ and 18/27 being CD56-. The Vγ2 chain sequences were determined and in each case, the phenotype (CD56 positive or negative) and the Vγ2 sequence of the clone matched what was found in the population analysis (data not shown). Unfortunately, none of these clones could be expanded to the levels needed for cytotoxicity assays. We repeated the cloning using IPP stimulation and plating on feeder cells. After multiple cloning efforts with PBMC from two different donors (N0001 and N0019), we obtained 15 clones that were phenotyped, had Vγ2 chain sequencing to confirm clonality and were used for cytotoxicity assays. Every clone expressed CD56 above the mean fluorescence intensity of 30, which is the usual cut-off for cell separations, suggesting that cells from the CD56+ subset had a higher cloning efficiency compared to CD56- cells. The clones all expressed NKG2D, a receptor required for Vγ2Vδ2 T cell cytotoxicity [21,22], with the percentage of NKG2D+ cells varying from 16% to 82% of cloned cells (Figure 7B). With the exception of clone 1-G10, all clones were cytotoxic for Daudi cells. Further, each of the CD56+ clones had a Vγ2 sequence that was matched to a sequence found in the CD56+ fraction of that donor's total Vγ2+ cell population. Despite representing approximately 50% of the Vγ2Vδ2 T cell population, the cloning studies produced few CD56- clones and they could not be expanded. Cloning studies showed that CD56 expression in clones was matched to the same Vγ2 clonotypes observed in purified sub-populations, and all of the CD56+ clones were cytotoxic for Daudi cell targets.

## Discussion

The CD56 cell surface glycoprotein is a marker for cytotoxic Vγ2Vδ2 T cells [17]. Within the circulating T cell population, individual Vγ2Vδ2 T cells fell mostly into two groups: precursor CTL effectors that express CD56 upon activation and cells that do not express CD56. For each human donor, cells identified by Vγ2 sequences mostly showed the same propensity for CD56 expression in samples collected 1 year apart, arguing that this property is stable. Thus, CD56 is not regulated stochastically on Vγ2Vδ2 T cells, but reflects a stable phenotype of individual cells in the population.

The next step was to discern whether CD56 is expressed on cells with unique TCR specificities. There was no fixed relationship between individual public Vγ2 chains and CD56 expression. Further, some of these chains defined populations present in both CD56+ and CD56- fractions. Individual nucleotypes showed a trend for appearing in CD56+ or CD56- fractions but not both, arguing that the capacity for CD56 expression was a pre-existing feature of individual cells i.e., they were precursors.

The analysis of nucleotypes provided the surprising result that unrelated donors express identical nucleotypes. Such events are known for NKT cells where the invariant Vα24 chain is the germ-line sequence without deletions or N-region additions [8]. For Vγ2 chains, conserved CDR3 regions may be due to limited deletion during recombination [23] and strong selection for these sequences since most of the N-region additions in these examples only affect the wobble base and do not change the amino acid sequence. There is strong selection for leucine, valine or isoleucine at position 97 in the Vδ2 chain CDR3 region and the conserved residues are believed important for phosphoantigen recognition [24,25]. In our study, the appearance of identical nucleotypes among unrelated donors complicates the analysis. If a sequence can arise in multiple donors, it seems likely that the same sequence can arise more than once in a single donor. For public nucleotypes, the individual Vγ2 chain sequence does not necessarily identify a single clone and complex properties must be expected.

The analysis of Vγ2 chain sequences showed overall, that individual clones of Vγ2+ cells in PBMC either had or did not have the capacity to express CD56 and become cytotoxic effectors. Whether this defines a lineage is unclear. Among T cells, lineage generally defines events during early cell differentiation. Recent studies in the mouse showed the capacity for IL-17/IL-23 expression defined a γδ T cell lineage [26]. The patterns for thymic CD27 expression and engagement with its ligand CD70, dictated entry into a cytotoxic γδ T cell lineage in mice [27], arguing that early events dictate the long-term outcome. Therefore, in the mouse, γδ T cell cytotoxicity is lineage-restricted. The human Vγ2Vδ2 repertoire contains few if any, naïve cells as it is the product of extensive extrathymic selection [18]. The CD56 marker is rarely expressed in human cord blood γδ T cells (Cairo et al, unpublished) and presumably reflects a subsequent developmental stage. Our studies cannot explain whether the capacity for CD56 expression arose early or late, and consequently, we hesitate to describe precursors to the CD56+ subset as comprising a lineage.

There was an intriguing trend between CD56 expression and clonotype frequency. Presumably the result of strong positive selection, the more abundant clonotypes tended to express CD56. This may reflect the increased apoptosis-resistance of CD56+ Vγ2Vδ2 cells [17], a trend also seen in CD56+ αβ T cells. The result of selection is an oligoclonal population of tumor effector cells that are stably maintained in the CD56+ fraction.

Whether Vγ2Vδ2 T cells belong to innate or acquired immunity is an enduring question in this field [28]. With a high proportion of cells expressing the Vγ2-Jγ1.2Vδ2 TCR there is an innate like response to phosphoantigen. However, there is clear evidence for selection and amplification of discrete subsets, including the Vγ2-Jγ1.2 rearrangement [14-16]. Our studies reported here show that chronic selection produces a population with phosphoantigen-specific TCR, but other mechanisms, likely operating before peripheral selection, dictate the potential for cytotoxicity and CD56 expression. Thus, the Vγ2Vδ2 TCR is necessary for tumor cell cytotoxicity because cell activation by antigen is required to develop effector function. However, cytotoxic and non-cytotoxic cells both proliferate in response to phosphoantigen. Owing to the greater complexity in αβ TCR repertoire, it is difficult to perform the same tracking studies, but it would be interesting to learn whether epitope-specific CD8 αβ T cells also display differences in CD56 expression that control cytotoxicity.

## Conclusion

Prior to selection and amplification mechanisms that shape the adult γδ T cell repertoire, individual clones are committed to the cytotoxic or non-cytotoxic lineages. Lineage commitment was not related to TCR specificity, as cytotoxic and non-cytotoxic precursors responded similarly to phosphoantigen stimulation. The mature population of Vγ2Vδ2 T cells includes committed precursors capable of expressing CD56 and cytolytic effector activity against tumor cells.

## List of Abbreviations

TCR: T cell receptor; CDR3: complementary determining region 3; NK: natural killer cell; NKT: natural killer T cell; KIR: killer immunoglobulin-like receptor; NKR: natural killer receptor; IPP: isopentenyl pyrophosphate; PBMC: peripheral blood mononuclear cells; CTL: cytotoxic T lymphocyte; FITC: fluorescein isothiocyanate; PE: phycoerythrin; APC: allophycocyanin.

## Authors' contributions

EMU generated the cell lines, obtained and aligned sequences, generated and tested clones, and wrote the manuscript. HL participated in obtaining and aligning sequences. CA participated in obtaining and aligning sequences and in testing clones. CF participated in obtaining and aligning sequences. CC helped to design and interpret the study results. CDP conceived of the study, participated in its design and coordination, and helped to write the manuscript. All authors read and approved the manuscript.
